# “What kind of support do I need to be successful as an ethnic minority medical student?” A qualitative study

**DOI:** 10.1186/s12909-020-02423-8

**Published:** 2021-01-05

**Authors:** Ulviye Isik, Anouk Wouters, Gerda Croiset, Rashmi A. Kusurkar

**Affiliations:** 1 Research in Education, Faculty of Medicine, Amsterdam UMC, Vrije Universiteit, P.O. Box 7057, 1007 MB Amsterdam, the Netherlands; 2 LEARN! Research Institute for Learning and Education, Faculty of Psychology and Education, VU University Amsterdam, Amsterdam, the Netherlands; 3 University Medical Center Groningen, Groningen, the Netherlands

**Keywords:** Academic performance, Ethnic minorities, Medical students, Motivation

## Abstract

**Background:**

To be in alignment with the increasing diversity in the patient population, ethnic minorities should have appropriate representation in health care professions. Medical students from ethnic minorities therefore need to be successful in their medical studies. The current literature highlights that they underperform in comparison with the ethnic majority. The aim of the present study is to gain insight into what medical students from ethnic minorities experience during their education and what they need to become or stay motivated and to perform to their full potential.

**Methods:**

Medical students from ethnic minorities from year 1 to 6, enrolled at Amsterdam UMC, Faculty of Medicine, Vrije Universiteit, the Netherlands, were invited via email to participate in this study. Semi-structured interviews were conducted, using an interview guide, from August–October 2018. A constructivist paradigm was adopted.

**Results:**

Eighteen medical students from ethnic minorities (three from year 1, three from year 2, one from 3, four from year 4, two from year 5, and three from year 6) participated in this study. Students’ negative experiences could be categorized as follows: (1) the effect of discrimination (2) lack of representation of ethnic minority role models, (3) lack of a sense of belonging, (4) lack of a medical network, (5) differences and difficulties in cultural communication and language, and (6) examiner bias in clinical assessments. Examples of support tips relating to these experiences are: increasing awareness about diversity and other religions, providing support groups, having visible ethnic minority role models, and facilitating support in networking.

**Conclusions:**

Findings of this study suggest that medical students from ethnic minorities have negative experiences that influence their education. Supporting these students is essential for creating a good and safe educational and practical environment for ethnic minority students.

**Supplementary Information:**

The online version contains supplementary material available at 10.1186/s12909-020-02423-8.

## Background

The patient population is becoming increasingly diverse. To ensure equitable healthcare, ethnic minorities should have appropriate representation in the health care professions. It is therefore essential that medical students from ethnic minorities are successful in their medical study. However, medical students with an ethnic minority background compared to the majority not only receive lower scores on knowledge and skills assessments in preclinical and clinical education, but also have difficulties in qualifying for specialty training [1–3]. Since motivation has been shown as a crucial factor for learning and academic success [4, 5], and could be a factor associated with underperformance of these students, the present study aims to investigate what support tips ethnic minority students have in order to sustain their motivation and perform optimally in the medical study. In the Netherlands, where this study is conducted, the percentage of medical students with an ethnic minority background is around 27%, and ethnic minority is defined as (Statistics Netherlands) [6]: “a person born in- or outside the Netherlands with at least one parent born outside the Netherlands”.

In this study the Self-determination theory (SDT) was used as the theoretical framework [7, 8]. According to SDT, motivation can be based on genuine interest (intrinsic motivation) and finding an activity personally important (identified regulation), which together form autonomous motivation. Motivation can also be driven by internal pressure, such as feelings of shame or guilt (introjected regulation), or external pressure or rewards (external regulation), which together form controlled motivation. Autonomous motivation is considered the most desirable type of motivation, because it is positively associated with well-being and academic success [4]. Fulfilling three basic psychological needs fosters autonomous motivation: autonomy (making one’s own choices), competence (feeling of capability) and relatedness (feeling of belonging to others) [9].

Ethnic minority students experience specific barriers in medical education. An insight into the barriers, how these affect students’ motivation, and what support they might need is necessary in order to arrange support systems that could aid the optimal performance of ethnic minority medical students. Previously identified barriers can be categorized into barriers related to: a) students themselves, b) educators/assessors and c) the learning environment. A barrier related to students themselves is that ethnic minority students and physicians were found to have less understanding of what it means to enter into specialty training and were less aware of the importance to start preparing for specialty training early in their education [3]. An explanation for these findings could be that ethnic minority students are often the first in their family to go to university or to study medicine [3]. A barrier related to educators/assessors is that medical students from ethnic majorities were more likely to receive a higher grade for their clerkship performance compared to ethnic minority students [2]. Also, despite the small differences in assessed clerkship performance between ethnic minority (underrepresented in medicine) and majority students, ethnic minority students received about half as many honors grades as compared to the majority students [10]. Barriers in the learning environment, which we found in an earlier study, include a lack of role models (e.g. specialists with and ethnic minority background) or a medical network, having the feeling of being ‘the other’ due to their ethnic background, miscommunication, and remarks about their appearance (e.g. wearing a head scarf) and accent (Isik U, Wouters A, Verdonk P, Croiset G, Kusurkar RA: As an ethnic minority, you just have to work twice as hard. Experiences and motivation of ethnic minority students in medical education: a focus group study, submitted). The ethnic minority students regularly experienced discrimination and cultural distance. Sometimes these experiences influenced their motivation negatively (Isik U, Wouters A, Verdonk P, Croiset G, Kusurkar RA: As an ethnic minority, you just have to work twice as hard. Experiences and motivation of ethnic minority students in medical education: a focus group study, submitted). Recurrent negative experiences can hamper student motivation and ultimately their long-term academic success.

What still remains to be investigated is what support these students need to stay motivated so that we can make recommendations for creating a support system for them. Thus, the aim of this study is to gain insight into what support medical students from ethnic minorities need in their learning environment to mitigate experienced barriers, sustain their motivation and ultimately perform to their full potential. The findings may be used to create a support system to facilitate the study success of ethnic minority students. The following research question guided this study:

What barriers do ethnic minority students experience in the learning environment and how can support structures be organised for these students in the learning environment in order to sustain their motivation?

## Methods

### Study design

This qualitative study intended to capture the experiences and needs of ethnic minority students in the medical curriculum. A constructivist paradigm was adopted, in which data and analysis are based on the interaction of the experiences of both respondents and researchers [11–14]. Constructivism states that everyone constructs their own reality by their own individual, social, and historical context, meaning that there is no objective reality [13]. Our aim with this approach was to understand the complex world from the point of view of those who live it, that is, the subjective experiences of ethnic minority students during their medical study [13].

### Participants and procedure

Medical students from ethnic minorities from all 6 years of medical study, enrolled at Amsterdam UMC, Faculty of Medicine, Vrije Universiteit, the Netherlands, were invited per mail to participate in this study. In this medical school approximately 30% of the students are from ethnic minorities. The first 3 years of the medical study form the preclinical education and the last 3 years form the clinical education [15]. In this study the definition of ethnic minority was in alignment with Statistics Netherlands [6]: “a person born in- or outside the Netherlands with at least one parent born outside the Netherlands”. The preclinical students were invited by email via the student administration department and clinical students were invited directly by the first author UI. The students who were interested in participating in this study approached the researcher via email. Because of the sensitivity of the study topic, snowball sampling was used to gather students [16, 17]. Interviews were conducted until we had gathered sufficient, appropriate and rich data to answer the research questions, i.e. when we reached data sufficiency [18].

### Data collection and analysis

Semi-structured interviews were conducted at Amsterdam UMC, Faculty of Medicine, Vrije Universiteit, the Netherlands in the period August–October 2018. Based on Malterud et al. (2015) [18], sufficient information power on the sample size for qualitative study depends on a few dimensions like the aim of the study (a broad study aim requires a larger sample size than a narrow aim), sample specificity (less large sample is needed with participants holding characteristics that are highly specific for the study aim), use of established theory (limited theoretical perspectives would usually require a larger sample to gather sufficient information) etc. After the 16th interview, we had enough data to answer our research questions and interviewing more students did not generate any new information. Based on these dimensions, we believe that we acquired sufficient data with 18 participants to answer the research questions. The aim of this study is specific; the needs of medical students from ethnic minorities in their education to become or stay motivated and to perform to their full potential. The characteristics of the participants are highly specific for our study aim; medical students from ethnic minorities. Further, we built on a qualitative study with a theoretical framework [(Isik U, Wouters A, Verdonk P, Croiset G, Kusurkar RA: As an ethnic minority, you just have to work twice as hard. Experiences and motivation of ethnic minority students in medical education: a focus group study, submitted), [18, 19].

The primary investigator (UI), with an ethnic minority background, performed the face-to-face interviews using a semi-structured interview guide (see Additional file 1). The development of the questions was based on our knowledge from previous study addressing ethnic minority medical students’ experiences [(Isik U, Wouters A, Verdonk P, Croiset G, Kusurkar RA: As an ethnic minority, you just have to work twice as hard. Experiences and motivation of ethnic minority students in medical education: a focus group study, submitted), [20], and the aim of the current study. Immediately after the interviews the interviewer wrote memos to capture her observations [21]. Data were audiotaped and transcribed verbatim. Braun and Clarke’s six stage of thematic analysis, a method that focuses on identifying patterned meaning across the dataset, was used to code and analyze the data [18]. The six stages are: 1) familiarizing with the data, 2) generating initial codes, 3) searching for themes, 4) reviewing themes, 5) defining themes, 6) writing-up [22]. First, UI read and reread the interviews and familiarized herself with the data. Then she coded all interviews using Atlas.ti. Examples of codes are ‘learn to be assertive’ and ‘being the other’. Next, codes were collated into potential themes, e.g. ‘support need: role models’. The second researcher (AW) followed the same steps and coded two interviews in order to discuss and develop our understanding of the data further. After that, the differences in the coding were discussed until consensus was reached. The discussions were mainly about the wording of the codes. Because as the understanding of the data was pretty much similar, he first researcher coded the remaining interviews. UI and AW checked and reviewed the themes. Next, UI refined and named the themes. Finally, a selection of illustrating quotes related to our analysis was made to report the findings and answer our research question.

Through iterative discussion the research team reached consensus about the findings. This contributed towards reaching our final insights into what medical students from ethnic minorities need in their learning environment to sustain their motivation and perform to their full potential.

The consolidated criteria for reporting qualitative research (COREQ) checklist was used to guarantee good quality [23].

### Reflexivity

The influence of the researcher is inevitable in qualitative research, which makes reflexivity crucial [24, 25]. In this study the ethnic minority background of the main researcher helped gain good understanding of the research topic and experiences of the participants. It likely also helped the participants in opening up and sharing their experiences and support tips [26]. However, being aware that researcher engagement might influence the data collection, e.g. during the interviews, the researcher aimed to use her background and own experiences merely as a lens to obtain understanding of participants experiences. In addition, the analyses were regularly discussed within the research group to make use of a variety of perspectives.

### Ethical approval

Participants were informed that participation in the study was voluntary, that their data would be handled confidentially, and that (non-)participation would not have consequences for their study. Written informed consent was gathered from all participants prior to conducting the interviews. This study was approved by the Ethical Review Board of the Netherlands Association for Medical Education (NVMO-ERB, dossier no. 2018.5.7).

## Results

Eighteen students participated in this study (11 female, 7 male), 7 from preclinical years (years 1–3), 9 from clinical years (years 4–6) and two in exceptional situations. Three were in year 1, three students in year 2, one in year 3, four in year 4, two in year 5, and three in year 6 of medical school. Preclinical education is year 1–3 and clinical education is year 4–6 at this medical school. One student had finished his preclinical education and was waiting to start with his clinical education. Another student was doing her clinical education, but decided to stop for a while. The respondents reported the following for their parents’ country of birth: Afghanistan, Armenia, Egypt, Ghana, Philippines, Morocco, Nigeria, Russia, Syria, Turkey, Ukraine, and Uzbekistan. One student reported that one of his parents was born in the Netherlands, however he was raised with the culture of his mother’s ethnic minority background. The interviews lasted 31–65 min.

We will present the different types of barriers students experienced, together with the support tips they described. Students’ experiences could be categorized as follows: (1) experiences of discrimination (2) lack of representation of ethnic minority role models, (3) lack of a sense of belonging, (4) lack of medical network, (5) cultural communication differences and language difficulties, and (6) and examiner bias in clinical assessments (see Table 1).

**Table 1:** Influencing factors and the support tips of medical students from ethnic minorities

Influencing experiences	Support tips
Experiences of discrimination	• Creating more awareness about diversity and other religions among all students and staff • A support group with students to discuss experiences of discrimination
Lack of representation of ethnic minorities and role models	• Role models (with an ethnic minority background)
Lack of a sense of belonging	• Voluntary (instead of mandatory) participation in physical examination as a subject for peers to practice on • More inclusive introduction days (at the beginning of the study) by making them attractive for a diverse group of students • Understanding for each other (ethnic minorities and ethnic majorities)
Lack of medical network	• Guidance in networking
Cultural communication differences and language difficulties	• Guidance in communication skills and overcoming language barriers. • Training to be assertive
Examiner bias in clinical assessments	• Assessors should communicate in time with students about their performance • Grading system for marks should be changed (for clerkship performance)

### Experiences of discrimination

Students experienced or had heard stories of friends who experienced demotivating discriminating comments related to their ethnic background in different educational settings, such as in a study group or during their clerkships:

“Yes, I know a Dutch majority medical resident. He said: ʿFatima [name changed for anonymity] sorry, but yes, your name is Fatima, and maybe you don't speak Dutch completely correctly so you’re ten nil down. [...] You will always have to work 10 times harder than a Dutch Freek, Joris [Dutch names for males] or whoever. It does not matter if they make the same mistakes, as a foreigner you will have a disadvantage and it will always stay that way. ʾ I did not believe him, but it turns out that's just the way it works.” (S1, female)

This student expressed that ethnic minority students are disadvantaged and always have to work harder to get the same opportunities as the ethnic majority students. This student expressed that she had almost stopped with her education at the department, but she continued, even though it was very difficult for her.

The students find their own way of coping with such experiences and continuing what they were doing:

“Yes, just answer and smile, stupidly smile. You do not even enter into the discussion. Yes, sometimes you dwell on it and sometimes you don’t. That's how my friends deal with it. Also, I know several people, [ethnicity] people who have failed an exam. Yes, how do they deal with it? As I said, just keep going on.” (S1, female)

Further, one of the students explained that she almost wanted to quit her education because of discriminating experiences during her clerkship, but she got over it, and accepted it as it is. This student also explained that sometimes students, including herself, pretend to be someone they are not and conform to the majority group to be accepted and to get into a specialty training:

“And sometimes I even go along with their conversation for fun and then I think ‘yes, that is what you want to hear right? That is not what I think...’ Yes, but what else should I do, it is all a game, unfortunately. It sounds very manipulative what I'm saying now, but that's how it works. [...] So if they [colleagues from ethnic majority] make racist remarks, ‘yes, there are those street youths around these days, surely many of them are Moroccans’. Yes, that’s just what it is, I agree with them.” (S1, female)

“[ … ] “fake it until you make it’ that’s everyone’s advice...” (referring to get into a specialty training; S1, female)

#### Support need

Students’ support tips in this domain pertain to the creation of more awareness about diversity and other religions to reduce such discriminatory comments. There is also a need for support groups to discuss with other students how to handle such situations:

“[ … ] sometimes you get remarks that may not be meant to be racist or discriminatory, but I am not sure how to interpret those. [ … ] How to deal with someone who says something about you, or does something against you. Do you take it personally that you look like that, or because it is something you do, or whatever... So how do you deal with it?” (S2, female)

Moreover, a regular one-on-one guidance with a counsellor was advised because this might be a safer setting to discuss (negative) experiences. A platform to talk about the prejudices that exist was also mentioned to help students increase their chances of being successful.

### Lack of representation of ethnic minorities and role models

Students explained that the medical doctors usually are White and male, and that there is a lack of a representation of ethnic minorities in the medical field. A student expressed her doubts of becoming a resident because of her ethnic background. She explained however that she could understand the mechanism, because ‘like attracts like’:

“[ … ] At that moment I could no longer imagine, ‘oh in the future I will also be a resident’. Because I saw only ethnic majority people around and that surprised me. [...] yes, they are still people from the study programme committee, they are usually Dutch people, from the previous generation, of the older generation, regardless, they choose people who they trust the most, or in whom they then have the most confidence, and I can also imagine, that ‘like attracts like’, from that principle.” (S3, female)

Further the students mentioned that they lack role models with an ethnic minority background:

“[...] you have no example. You have neither someone who can mentor you, nor an example to work towards. Because even if you are doing an internship in your first year and in hospitals where you only see White doctors and then you think: Ok, but will I ever get there?” (S4, male)

#### Support need

Different students expressed how role models (from ethnic minorities) can be supportive and motivating:

“Well, I think that if he [a specialist with an ethnic minority background] can do it, thus as an ethnic minority, then it would also be possible for other ethnic minorities or me to succeed.” (S5, female)

Senior students or specialists were seen as role models and could serve as a role model in different ways. They can teach, coach, help to support the students (as a mentor/teacher) based on their own experiences in their path to become a doctor. For example, a student explained that that if he had a supervisor (with an ethnic minority) background with whom he could talk about his ethnicity related experiences that negatively influence his motivation, he might know how to handle in such situations.

### Lack of sense of belonging

Students expressed that they lack a sense of belonging to the medical environment. A student even mentioned that she knew students who dropped out of the medical study, because they felt that they did not belong to the medical doctor group. Students also explained the experienced difficulties of ethnic minority students during their physical examination, because of their religion. The notion that they have might have to undress during practical sessions caused unease.

Moreover, ethnic minority students experienced less belonging to majority students because of different interests:

“Because they try to do that, they try to organize something in a way that makes the whole group more connected, these are the introduction days to get to know each other, but ethnic minority groups really feel left out. I went there once, I had booked for 2 days, and the first day I was there and there was alcohol everywhere again and I did not feel comfortable at all, and then the second day I also did not go.” (S6, female)

A consequence is a division between ethnic minority and majority students:

“The majority group has that feeling of belonging together because they have had introduction days together, so then they know each other a bit, but an ethnic minority group. if they are Muslim for example, or religious or Christian or whatever also, then they try it, but then they feel a bit weird. And then they do not go anymore, and then they come back to the lecture hall, and then the majority group usually have people they know from the introduction days, and the minority group is like, I don’t know anyone yet so I go and look for the minority group.” (S6, female)

A participant told that it is a pity that there is sometimes no click between ethnic minority and majority students. Another student expressed that as an ethnic minority, you should be very intrinsically motivated to cross the finish line. Having others in your surroundings who can join you in the conversation, think along with you, and help you, makes it easier.

#### Support need

Giving students the choice to be a subject during physical examination or not was mentioned as a solution for religious ethnic minority students:

“Yes, if you want it then, there are usually other girls who want to do it. I never took off my pants and I've always had the opportunity to practice, and other students too, so it should be voluntary, I think. [...] So in my practical lessons I really never had problems with it.” (S5, female)

The introduction days can be made attractive for all students by separating the informative part from the social activities, and/or making the social activities interesting for all students, such as gymnastics:

“[...] Often also something playful (referring to gymnastics), just doing something fun. [ … ] But just doing something nice, if you take that aspect and thereby connect ethnic groups with the majority, that helps.” (S6, female)

Another support tip was creating more understanding for each other (ethnic minorities and ethnic majorities):

“Because they will be colleagues later on, the future doctors. And for that they have to have understanding and by them, I mean the ethnic minority students, and it is useful if ethnic minority students simply realize what the problems are, how do my peers, my co-ethnic minority students deal with it, what can I do to go through the study? And it would be nice if there is a good collaboration with the majority students.” (S7, male)

### Lack of medical network

The lack of medical network was expressed by different students. Students mentioned that ethnic minority students generally do not have a medical doctor as a parent:

“For example, Moroccans, Turks who start studying medicine, do usually not have a mother or father who is a doctor, do not have a network of doctors, so their information about the medical world is quite limited. And in most cases actually nil, nothing. While the ethnic majority students have a dad, mom, uncle, the parents of their boyfriend, girlfriend who are a doctor, and who sometimes explain things and you already have a network. The Moroccans, Turks and other ethnic minority medical students are 1-0 behind in that concern.” (S7, male)

A student expressed how lacking a medical network can be a problem in medical education:

“That there is always being emphasized that you have to network, and that it is very difficult because … For example, I do not have my family in the medicine world and in your second year you have to arrange a GP internship, for example, to name something very concrete. Well and then you hear from your Dutch majority friends that they found a doctor in no time, while I had to email all around Amsterdam and then randomly to find someone.” (S4, male)

Another student explained that students from ethnic minorities usually live in communities in which family is an important aspect and the need to connect with others is limited. Not learning how to make connections outside the community, together with hearing stories about ethnic minority students being less likely to get hired because these students to believe that they will not succeed. Ethnic minorities find networking outside of their comfort zone, and ethnic majority students see it as a more natural thing.

#### Support need

Students wished to be facilitated in building and maintaining a network and suggested that this should include help from an ethnic minority (role model). Different ways concerning guidance in networking were mentioned: conducting workshops, networking events, lectures, receiving tips and help from senior students etc. Another idea was involving ethnic minority students more in research that is related to their identity/ethnicity, than they are more willing to help with the research and in this way, they can build their network. However, a few ethnic minority students expressed that you have to take matters into your own hands and build your own network.

### Differences and difficulties in cultural communication and language

Cultural communication differences between the ethnic majority group and minority groups include misinterpretation or misunderstanding by someone from the ethnic majority group. Sometimes ethnic minorities do not respond react because they think: ‘never mind, he did not understand my question’.

“And I had that feeling, and then I asked fellow students, and I have experienced it also that you just do not get the answer to your question. You get an answer to another question, but that is how that person interpreted it.” (S7, male)

This student explained this is because of the different religious and cultural values he has with the majority group.

Assertiveness was also mentioned. One student explained the differences in being assertive between the students from ethnic majority and ethnic minorities as follows:

“[ … ] I think that ethnic majorities are certainly more assertive. But that is because the circumstances make it easier for them to be assertive. [ … ]as an ethnic minority student, you have many more challenges... yet many more obstacles. And that it is much more difficult to be assertive. So, I think that ethnic majority students are more assertive, but that is also because they have grown up in an environment where they are expected to, and in which it is stimulated. While ethnic minority students are often told, you know the bigger they are, the harder they fall, try not to attract too much attention... [ … ]” (S4, male)

Language difficulties also influenced ethnic minorities during their education. A student mentioned his difficulty with writing an essay about physical examination:

“[..] it took me more time than a normal Dutch student, so I have the feeling that I … a kind of powerless feeling.” (S8, male)

#### Support need

Help in communication and language/writing skills is needed. Furthermore, role models can help with situations in which ethnic minority students encounter communications differences or are misunderstood:

“[ … ] So, if I ask a question for a certain purpose, then the ethnic minority supervisor, can understand the question from my perspective, from my cultural and religious value. And the ethnic majority supervisor does not understand that, because he does not know the value that I have. So, as a listener you have to understand where does a question come from. And then I can also answer him like that.” (S7, male)

Further, a few students expressed that for ethnic minority students it is important (to learn) to be assertive. One student even expressed that being assertive is the key message, also for networking.

### Examiner bias in clinical assessments

Students believed that there is subjectivity in the assessment of the clerkship performance. For this student it is an explanation to the differences in grades between ethnic minority and majority students:

“Well, because if you are not right up the alley of a specialist, or do not fit within a team, that is also immediately included in your grade. So yes, I never link it very much to the competencies that students have or do not have and that they because of that get lower marks, poorer study results, but I link it much more to the subjectivity of the assessment.” (S9, female)

This student based her perception about the subjectivity on the stories of other ethnic minority students like “you do not really fit into the team, or you are not really suitable for … or this does not really suit you [ …]”.

#### Support need

Students proposed several changes, i.e. timely and honest communication between supervisors and students about students ‘clerkship performance and changing the grading scale from 0-10 into ‘pass-fail’:

“[ … ] the way in which the study results are also collected in the clinical years, you cannot avoid subjectivity. You will work in a department; your performance will be assessed so there is simply an opinion of an assessor and the people who work there. The question is, do you want to put marks on that too? In the end, maybe we just have to go to a ‘pass-fail’ system and I think that is actually more relevant than grades. [...] by abolishing that you might actually have that difference...” (S9, female)

### Preconditions

Students also stressed some preconditions to the proposed support interventions. Students mentioned that support programs should not result in ethnic minority students being put away as a separate needy group. Interventions should therefore be offered to and aimed at the entire student population:

“Because there is perhaps a barrier that people have that kind of ‘oh, if I go there then maybe I am such a person who...’ you know, it is perhaps that you indirectly create a kind of separation between the large group that has a background different from Dutch, and people who don’t have. And if, you might get a split again.” (S10, male)

“...because the moment that you focus on this too much, I think then people will also, that the people will also recoil, not recoil, but that they think ‘forget about it because why are you focusing on this so much.’” (S11, female)

Another precondition was that interventions should be offered from the beginning of medical education:

“In the beginning of the year, so not that it is suddenly halfway through the year, when it is actually already a bit too late. When you are struggling a little, say in those first 2 months. Because those first few months are the hardest.” (S4, male)

## Discussion

**Fig. 1:**
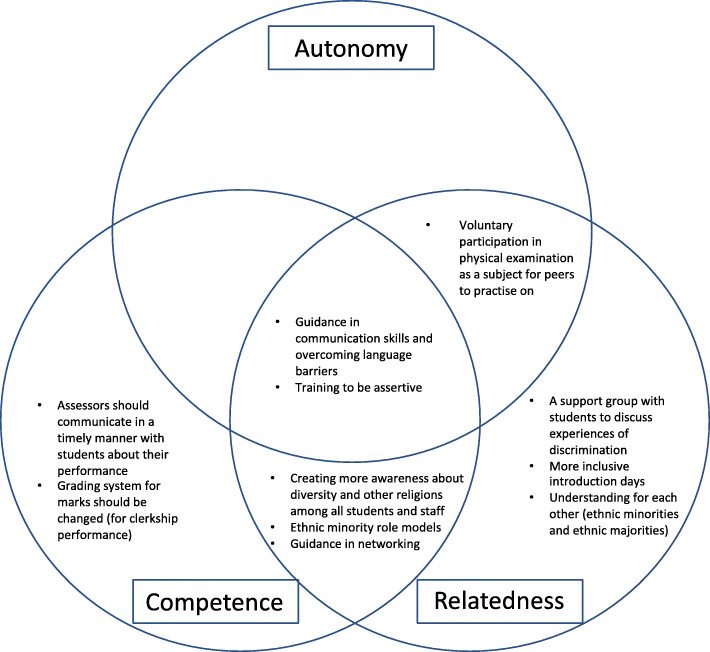
The support tips supporting autonomy, competence and/or relatedness of medical students from ethnic minorities

This study aimed to gain insight into what medical students from ethnic minorities need in their learning environment to sustain their motivation and perform to their full potential. The students in our study described various experiences influencing the education of ethnic minority students, such as discrimination, a lack of representation of ethnic minority role models, and a lack of medical network. In addition, students expressed support tips that could help ethnic minority students, such as creating more awareness about diversity and other religions, visible ethnic minority role models, and guidance in networking. We considered these findings through the lens of the Self-determination theory. Our findings seemed to support the autonomy, relatedness and/or the competence needs of ethnic minority students (see Fig. 1). This adds to the literature on this topic. We elaborate on this below.

Students expressed different discriminating experiences related to their ethnic background in the learning environment. Our finding that ethnic minorities often given a positive twist to discriminating experiences in order to persist with their education to become a doctor is supported by an earlier study (Isik U, Wouters A, Verdonk P, Croiset G, Kusurkar RA: As an ethnic minority, you just have to work twice as hard. Experiences and motivation of ethnic minority students in medical education: a focus group study, submitted). In our study, one student mentioned that she almost stopped with her education. We find it important to support every student to perform optimally through creating awareness among students and staff about such experiences because discrimination can lead to an unsafe and stressful environment that causes more vulnerability, stress, and mental and physical health problems [27]. In addition, this would help students’ feelings of relatedness and competence.

Our findings align with studies among ethnic minority undergraduate students [(Isik U, Wouters A, Verdonk P, Croiset G, Kusurkar RA: As an ethnic minority, you just have to work twice as hard. Experiences and motivation of ethnic minority students in medical education: a focus group study, submitted), [28] and residents [29], in which the issues of ‘lack of a sense of belonging’ and ‘being the other’ have been identified. This indicates that ethnic minorities struggle with their sense of belonging throughout the entire medical education continuum. Research has shown that the involvement of minority faculty and staff increased students’ sense of belonging, and matured in their sense of responsibility to ethnic majorities and minorities on the campus [30]. In addition, mentors/counselors with intercultural competencies or an ethnic minority background could help in building trusting relationships with ethnic minority students, understanding him/her better, and therefore supporting the student adequately. Teachers with an ethnic minority background can contribute to a positive image of the study program and give students more confidence and increase their feelings of relatedness [31]. In this way the students can feel more autonomous and related in the learning environment.

The current and previous studies have emphasized the lack of ethnic minority role models and its effects [(Isik U, Wouters A, Verdonk P, Croiset G, Kusurkar RA: As an ethnic minority, you just have to work twice as hard. Experiences and motivation of ethnic minority students in medical education: a focus group study, submitted), [32, 33]. Furthermore, ethnic minority residents reported that they have a less relevant academic network, usually do not have much experience in networking and lack role models [3]. To reduce future educational and professional gap between ethnic majority and minority students, ethnic minority students could benefit from help with improving their skills to build and maintain networks, and seeing ethnic minority role models from early on in their education. Moreover, role models from ethnic minorities could be crucial as their influence is through the hidden curriculum rather than the formal curriculum. The hidden curriculum refers to “processes, pressures and constraints which fall outside of, or are embedded within, the formal curriculum, and which are often unarticulated or unexplored” [34]. Lack of role models in the hidden curriculum could create a feeling of incompetence, i.e. that the ethnic minority student may feel not be able to achieve to his/her potential because he/she doesn’t see any successful cases of people similar to him/her around in the learning environment. Visible role models from ethnic minorities, could help ethnic minority students realize that their dream and goal of becoming a doctor is feasible [35], and could stimulate their feelings of relatedness. However, there were a few ethnic minority students expressing that you have to take matters into your own hands and build your own network, so this might not work for all ethnic minority students.

The students who are from non-ethnic minority background who are first generation students have similar difficulties in understanding the medical profession and lack the networks for internships. However, from previous research we know that medical students from ethnic minorities are more often first-generation students and less often have a medical doctor as parents [36]. This creates a higher disadvantage for them.

Language barriers and communication differences could also have negative consequences for ethnic minority students’ autonomy, competence and relatedness. Such aspects usually pertain to not being assertive and being misunderstood. Generally, ethnic minority students speak Dutch fluently, sometimes only with an accent, because they are born and raised in the Netherlands. However, there are also ethnic minority students that moved to the Netherlands and face problems with the Dutch language. In general, a high level of Dutch is required and all medical students are tested on their Dutch language level at the beginning of their education at this medical school. Ethnic minority students are aware that they should be assertive and advise other students to be assertive [31]. Specialists and heads of departments have pointed out that being assertive is crucial in profiling yourself for specialty selection [3]. However, being assertive can be difficult for ethnic minorities because of their cultural (collectivistic) background [3], (Isik U, Wouters A, Verdonk P, Croiset G, Kusurkar RA: As an ethnic minority, you just have to work twice as hard. Experiences and motivation of ethnic minority students in medical education: a focus group study, submitted)]. Uncertainty about language can also be an underlying factor why interactions with other students or teachers fail to appear (e.g. not daring to ask questions), in understanding the learning material and being able to follow the lessons [31]. These findings show that it is crucial that medical schools should train students early in the education on how to be assertive and offer help in majority language to remove these barriers. Medical students who receive an assertiveness training have been found to have significantly improved in their assertiveness and self-esteem compared to those who did not receive it [37].

Examiner bias in clinical assessments was also expressed. There was a need for better, honest, and timely communication about students’ clinical performance. Moreover, it was mentioned that subjectivity might be reduced by changing the clerkship grading system from a ‘0–10′ scale into ‘pass-fail’. Another quantitative study also identified the subjectivity among assessors based on students’ ethnicity. The use of several assessments performed by different assessors at various moments decreases the variation in clinical grades based on student’s ethnicity [38]. Changing the grading system, as explained in this study, could support the need for competence of ethnic minority.

### Limitations

A limitation of this study was the used definition of an ethnic minority. The definition of Statistics Netherlands does not include the third-generation ethnic minorities: a person born in the Netherlands, and of whom the parents are also born in the Netherlands, but of whom at least one grandparent is born outside the Netherlands [6]. However, research has shown that the ethnic minorities from the third generation still experience discrimination based on their ethnicity [39]. These students and their experiences might have been overlooked in this study because of the use of the definition of Statistics Netherlands. Moreover, because of the sensitivity of the study topic in our qualitative studies snowball sampling was used to gather students [16, 17]. This might have led to the inclusion of more resilient or motivated ethnic minority students, who may feel more comfortable with their ethnicity in particular situations than the majority of ethnic minority students. However, a lot of negatively influencing experiences were identified. These experiences may even have more impact on the motivation of those ethnic minority students who are less resilient and motivated.

### Recommendations

All students will have their own struggles on their path to become a doctor. However, students from ethnic minorities also face the experiences described above, this shows that they could have more struggles than ethnic majority students.

Several recommendations for students and staff in medical education arise from students’ suggestions in our study. The students and staff should be aware that ethnic minority students struggle within the educational and practical environment based on above noted experiences. In Fig. 1 we present the recommendations based on the support tips indicated by students, and whether these recommendations support their autonomy, competence and/or relatedness.

### Further research

The findings indicate that students from ethnic minorities still have the need for support in their education. Follow-up research on the effects of such support programs in practice should be conducted to establish their usefulness in medical education, e.g. the effects of assertiveness training on medical students from ethnic minorities and the effects of culturally competency trainings, e.g. to create more awareness about diversity and other religions, on medical students and staff should be explored. Future research comparing the effects of clerkship assessments graded with a scale ‘0–10’ with ‘pass-fail’ is necessary to determine whether ethnicity plays a role in the assessments.

## Conclusions

The results of our study suggest that ethnic minority students face different situations that influence their education and (still) have need for (specific) support. Supporting ethnic minority students is essential to create a good and safe educational and practical environment in order for these students to be successful.

## Supplementary Information

**Additional file 1.** Interview guide.

## Data Availability

Anonymized data are available from the corresponding author on request.
