# Supplementary material for: “What kind of support do I need to be successful as an ethnic minority medical student?” A qualitative study
Source: BMC Med Educ. 2021 Jan 5;21:6. doi: 10.1186/s12909-020-02423-8 (PMC7786944; doi:10.1186/s12909-020-02423-8)
Supplement: Supplementary file 1 — Additional file 1. Interview guide. [file 12909_2020_2423_MOESM1_ESM.docx]

**Additional file 1. Interview guide**

| **Interview questions** | |
| --- | --- |
| 1. | In your ideal world, what would the medical education look like? |
| 2. | What are the aspects that influence your motivation and academic performance within the learning environment? |
| 3. | What should be done in the curriculum to help you to stay motivated during your education? |
| 4. | Do you have other ideas about what could help you stay motivated and perform better during the education? |
| 5. | If you could set up a support program yourself with the aim of motivating / encouraging students from ethnic minority groups, how would you do it? |
